# Longevity factor klotho and chronic psychological stress

**DOI:** 10.1038/tp.2015.81

**Published:** 2015-06-16

**Authors:** A A Prather, E S Epel, J Arenander, L Broestl, B I Garay, D Wang, D B Dubal

**Affiliations:** 1 Department of Psychiatry, University of California, San Francisco, San Francisco, CA, USA; 2 Department of Neurology, University of California, San Francisco, San Francisco, CA, USA

## Abstract

Chronic psychological stress is associated with accelerated aging and premature morbidity and mortality; however, the biology linking chronic psychological stress and its maladaptive effects remains largely unknown. Klotho is a pleiotropic hormone that regulates the aging process and promotes better brain and body health. Whether klotho is linked to psychosocial stress or its negative impact in humans has not been investigated. To address this gap, we recruited 178 healthy women who were either chronically high-stress maternal caregivers for a child with autism spectrum disorder (*n*=90) or low-stress control mothers of a typically developing child (*n*=88). We found that women under high chronic stress displayed significantly lower levels of the longevity hormone klotho compared with low-stress controls (*t*(176)=2.92, *P*=0.004; *d*=0.44), and the decrease among those under high stress was age-dependent. In addition, high-stress caregivers who reported more depressive symptoms displayed even lower klotho levels compared with low-stress participants. These findings provide the first evidence that klotho levels are sensitive to psychosocial stressors and raise the possibility that klotho may serve as a novel biological link connecting stress, depression and risk for accelerated disease development. Furthermore, these findings have important implications for understanding the plasticity of the aging process and may represent a therapeutic target for mitigating the deleterious effects of chronic psychological stress on health and well-being.

## Introduction

Improvements in medical care over the past century have enabled people to live longer than in any time in human history. With advancing age, however, comes increasing susceptibility to a multitude of chronic diseases of the body and brain. Whereas chronological age is the strongest predictor of disease risk, the rates at which individuals succumb to disease vary substantially. Accordingly, investigating novel biomarkers that may account for this variability could illuminate biological mechanisms that underlie plasticity of the aging process.

Klotho is an aging regulator and pleiotropic hormone primarily produced in the kidney and choroid plexus of the brain that circulates throughout the body following cleavage from its transmembrane form.^1, 2, 3, 4, 5, 6^ Well studied in model organisms (for example, mice^4^ and nematodes^7^), klotho is an aging regulator that, when overexpressed, extends lifespan^4, 7^ and, when disrupted, promotes aging phenotypes,^3^ including atherosclerosis,^8^ decreased bone mineral density^6, 9, 10^ and osteopenia.^11^ The effects of klotho are complex and multifaceted. Klotho regulates insulin Wnt, and *N*-methyl-D-aspartate receptor signaling,^4, 6, 12, 13, 14^ influences ion channel clustering and calcium homeostasis^15, 16^ and promotes FGF23 activation.^17, 18^ Klotho also provides protection against endothelial dysfunction,^19, 20^ suppresses oxidative damage^21^ and aids in regulating inflammatory activity.^22^ In humans, being heterozygous for KL-VS, a functional genetic variant in the *KLOTHO* gene, is associated with higher serum klotho levels,^12, 23^ longer lifespan,^23, 24^ lower rates of age-related disease (for example, cardiovascular disease)^25^ and better cognitive function.^12, 26^ Further, higher serum klotho levels prospectively predict better daily functioning (that is, activites of daily living) and lower mortality risk in older adults (aged 65 and older), independent of age, sex, body mass index (BMI), education and health status.^27, 28^ Despite the potential of klotho as a marker of biological aging, there is no research to date examining whether klotho levels relate to psychosocial factors (for example, chronic stress) well known to predict elevations in age-related disease risk.

Chronic psychological stress, such as the stress conferred by serving as a caregiver to a spouse with dementia or a child with a chronic illness, has consistently been associated with accelerated age-related disease and onset of psychiatric illness,^29^ chiefly major depression.^30^ A large literature demonstrates that chronically stressed caregivers report higher levels of distress and depressive symptoms and suffer more health problems, including cardiometabolic conditions^31, 32^ and premature mortality,^33, 34^ compared with non-caregivers (reviewed in Pinquart and Sorensen^29^ and Vitaliano *et al.*^35^). Despite the clear role of chronic stress in impairing physical and mental health, the mechanisms through which these deleterious effects are conferred remain to be fully elucidated.

Given the growing, and compelling, experimental evidence implicating klotho in aging and the strong link between stress and brain and body health, the aims of the present study were to investigate, for the first time, whether klotho, measured in peripheral circulation, differed in healthy, premenopausal women who were and were not experiencing chronic psychological stress using a well-validated caregiver stress model. We further examined whether klotho levels were associated with symptoms of depression, a common consequence of chronic psychological stress. We hypothesized that women under chronic stress would display lower levels of klotho compared with low-stress, age-matched controls, and that these differences would be greater in women concurrently reporting greater depressive symptomatology.

## Materials and methods

### Participants and procedures

Participants were 183 mothers recruited via schools, parenting publications, social media, mailings and ads through child development centers in the San Francisco Bay Area and direct recruitment at the University of California, San Francisco Autism Clinic; 178 of the mothers had serum samples available for analyses. Eligible participants were non-smokers between the ages of 20 and 50 years, with at least one child between the ages of 2 and 16 years. To be characterized as a high-stress maternal caregiver, the participant had to care for a child diagnosed with autism spectrum disorder and report a score of ⩾13 on the Perceived Stress Scale.^36^ Low-stress maternal controls were characterized as caring for a neurologically typical child and a reported Perceived Stress Scale score of ⩽19. The Perceived Stress Scale eligibility criteria were based on prior national norms^36, 37^ and facilitated analyses of continuous stress measure irrespective of the caregiver status. All participants reported being premenopausal and in good general health with no major diseases, including no history of coronary heart disease, endocrine disorders, epilepsy, brain injury, autoimmune conditions, severe asthma or lung disease. Potential participants were excluded if they had cancer or had undergone chemotherapy or radiation in the past 10 years. Structured Clinical Interviews for Diagnostic and Statistical Manual for Mental Disorders for Axis I Disorders (SCID) were carried out during the eligibility period and individuals with current psychiatric conditions, including bipolar disorder, post-traumatic stress disorder and eating disorders were also excluded. Low-stress maternal controls with current major depression were excluded; this was not exclusionary in high-stress maternal caregivers. All study participants were free from medications known to affect the immune and endocrine system with the exception of antidepressant medication and oral contraceptives. Two controls were taking antidepressants for reasons other than depression (premenstrual dysphoric disorder and sleep).

At the initial study visit to the laboratory, participants completed a battery of sociodemographic, psychological and health behavior questionnaires and underwent a fasting morning blood draw. BMI (kg/m^2^) was assessed during the initial study visit. Sociodemographic questions included age, self-identified race/ethnicity, household income and highest level of education. Levels of depressive symptoms were assessed using the Inventory of Depressive Symptomatology, a 30-item self-report measure of depression severity.^38^ This scale is widely used with strong reliability and validity.^38, 39^ Scores can range from 0 to 84 with categories of severity established as follows: 0–13=none; 14–25=mild; 26–38=moderate; 39–48=severe; 49–84=very severe.^40, 41^ Participants were paid $110 at the conclusion of the baseline assessment. This study was approved by the Institutional Review Board at the University of California, San Francisco, and written, informed consent was obtained for each study participant.

### Assay of klotho

Serum from morning fasting blood samples was collected and stored at –80 °C. Soluble α-klotho was measured using a solid-phase sandwich enzyme-linked immunosorbent assay (Immuno-Biological Laboratories, Takasaki, Japan),^42^ as described.^12^ Briefly, serum was diluted fourfold with the Immunoassay buffer and a standard curve was established by serial dilution of recombinant human α-klotho protein. Diluted serum was loaded in duplicate onto a plate pre-coated with affinity-purified anti-human klotho (67G3) mouse IgG monoclonal antibody. Controls were included as references for each plate to enable accurate interplate comparisons. The plate was incubated for 1 h at room temperature and washed seven times with washing buffer. Horseradish peroxidase-conjugated anti-human klotho (100 μl, 91F1) mouse IgG monoclonal antibody was added to the plate and incubated for 30 min at room temperature. Plates were then washed nine times and the reaction was visualized by the addition of 100 μl of chromogenic substrate for 30 min at room temperature. The reaction was stopped with 100 μl of 1 *n* H_2_SO_4_, the absorbance at 450 nm was measured on a Spectramax 190 plate reader (Molecular Devices, Sunnyvale, CA, USA), and α-klotho levels were calculated using the SoftMax Pro software (Molecular Devices). The assay had an average coefficient of variation of 3–5%. Samples with coefficient of variation above 10% were re-run.

### Statistical analyses

All analyses were carried out using SPSS version 22 (SPSS, Chicago, IL, USA). Data were assessed from 183 volunteers who participated in the baseline assessment of the study; of those, serum was available on 178 participants. Because this was the first study on stress and klotho, we relied on a prior lifestyle study (that is, physical activity)^43^ to estimate the sample size needed to detect a small to medium effect size. Pearson-moment product correlations, independent *t*-tests, *χ*^2^-test and analysis of variance with *post hoc* comparisons were conducted to examine associations between study covariates, levels of klotho and caregiver status. To examine differences in klotho between high-stress caregivers and low-stress caregivers we conducted independent *t*-tests and analysis of covariance. To identify covariates to be used in statistically adjusted models, bivariate associations between possible covariates and klotho were first calculated; variables significantly associated with klotho were retained in adjusted models. This strategy conserved power and increased degrees of freedom in multivariate models. As little is known about possible confounding factors, we used only those that manifest an association in this relatively small sample. To test whether the age-related declines in klotho differed between high-stress and low-stress caregivers, the sample was stratified based on the caregiver status and separate multiple linear regressions associating age with klotho were computed. Finally, a multiple linear regression model, adjusting for covariates, was computed to test associations between levels of depressive symptoms and levels of klotho. Effect sizes were calculated using Cohen's d for between group differences while adjusted *R*^2^ values are provided for regression analyses. Fisher's *Z*-test was used to test group differences in associations between high- and low-stress caregivers. The distribution of klotho was non-normally distributed and underwent log-10 transformation to approximate a normal distribution. Null hypotheses were rejected below a *P*-value of 0.05.

## Results

### Subject characteristics

Sociodemographic and psychosocial characteristics of the cohort (*n*=90 high-stress caregivers, *n*=88 low-stress controls) are shown in Table 1. As expected,^44, 45^ high-stress caregivers reported higher levels of perceived stress and greater depressive symptoms than low-stress controls. There were no significant group differences in age, BMI, racial composition or education level. High-stress caregivers were more likely to report household incomes below $100 000 than low-stress controls, and were more likely to be taking antidepressants. In the sample as a whole, lower levels of klotho associated with older chronological age (*r*=−0.16, *P*=0.029) as anticipated.^12, 42^ In addition, higher BMI scores (*r*=−0.15, *P*=0.048) were associated with lower levels of klotho. Other sociodemographic variables (education, income and race) and antidepressant use were not significantly associated with klotho levels (*P*-value>0.05).

### Klotho levels are lower in chronic, high-stress caregivers

**Figure 1:**
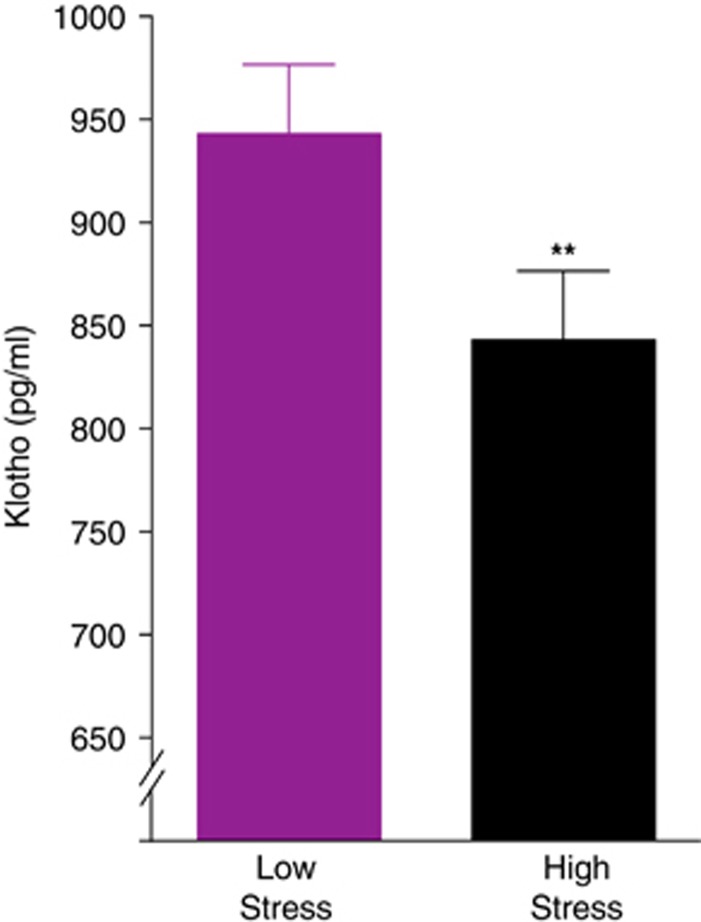
Klotho levels are lower in young women under chronic, high stress. Fasting morning serum klotho levels of high-stress maternal caregiver women (*n*=90) and low-stress controls (*n*=88) are shown. Analysis of covariance (ANCOVA) analyses revealed that high-stress women showed lower levels of klotho compared with low-stress controls (*t*(176)=2.92, **P=0.004). These differences remained significant after accounting for effects of age and body mass index (BMI; F(1, 173)=7.27, *P*=0.008). Analyses were carried out on log10-transformed klotho values. Data are means±s.e.m.

We hypothesized that high stress would be associated with lower levels of circulating klotho. We found that women with high chronic stress displayed lower serum levels (*M*=836.0 pg ml^−1^, s.d.=327.7) of klotho compared with low-stress controls (*M*=949.0 pg ml^−1^, s.d.=311.8) by 12% (*t*(176)=2.92, *P*=0.004; *d*=0.44). This difference remained statistically significant after adjusting separately for age (*P*=0.006; *d*=0.42), BMI (*P*=0.006; *d*=0.42), and after adjusting for these factors together (*P*=0.008; *d*=0.41; adjusted means displayed in Figure 1). Higher levels of perceived stress were statistically related to lower levels of klotho in bivariate (*r*=−0.11, *P*=0.04) but not age-adjusted (*r*=−0.10, *P*=0.17) analyses.

### Klotho levels show age-related decline in high-stress caregivers

**Figure 2:**
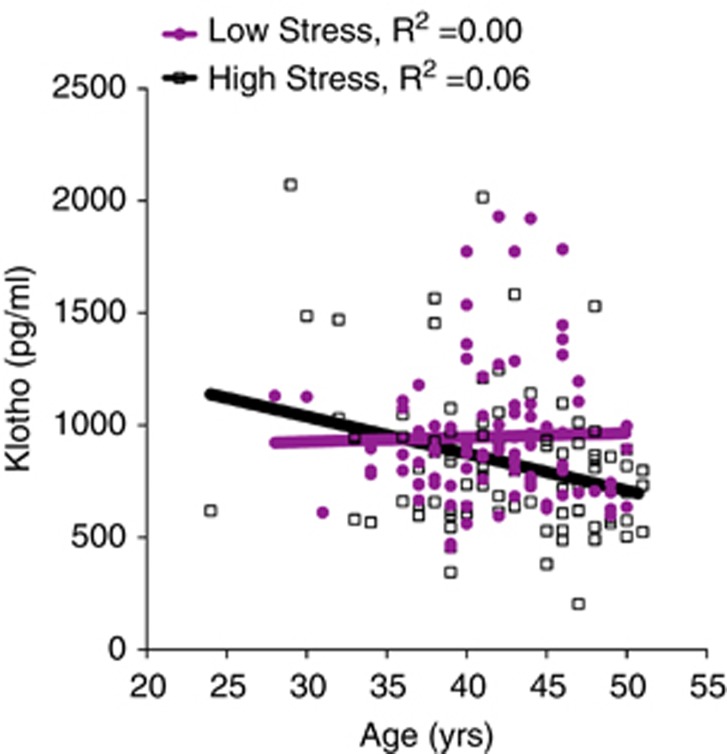
Klotho levels show age-related decline in young women under high, chronic stress. Klotho levels decreased with increasing age in chronic, high-stress caregivers (linear regression *B*=−0.26, *P*=0.014; *R*^2^=0.06), but not low-stress women (*B*=0.01, *P*=0.934; *R*^2^=0.00). This association remained significant after accounting for body mass index (BMI; *B*=−0.26, *P*=0.016; Δ*R*^2^=0.05). Analyses were carried out on log10-transformed klotho values.

As chronic psychological stress is associated with accelerated biological aging,^44, 46, 47^ we explored whether age-related decline in klotho occurs more rapidly under high stress. We found that high-stress women (*B*=−0.26, *P*=0.014; *R*^2^=0.06), but not low-stress controls (*B*=0.01, *P*=0.934; *R*^2^=0.00), showed a significant age-related decline in klotho levels (Figure 2). The association between low klotho and age among high-stress caregivers remained significant after adjusting for BMI (high stress: *B*=−0.26, *P*=0.016; Δ*R*^2^=0.05; low stress: *B*=0.03, *P*=0.815; Δ*R*^2^=0.00). There was a trend for a significant group difference in these associations (*Z*=1.87, *P*=0.06). Thus, even among young otherwise healthy women, those under chronic psychological stress, but not under lower levels of stress, showed accelerated age-dependent decline in klotho.

### Lower klotho levels are linked with depressive symptoms

**Figure 3:**
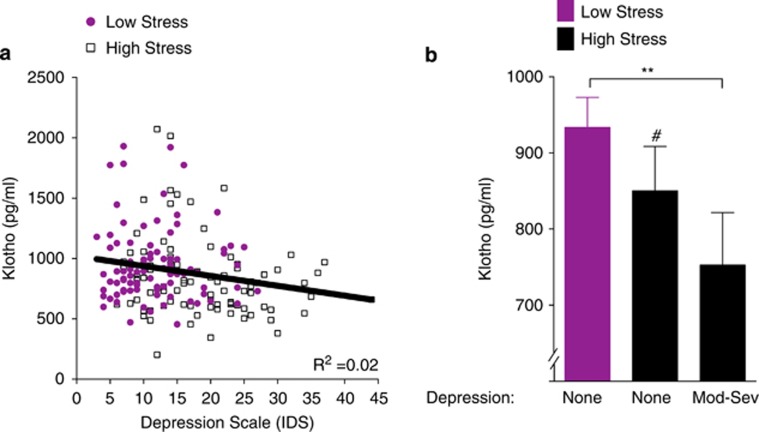
Lower klotho levels are associated with clinically significant depressive symptoms in chronically high-stress women. (**a**) In all women assessed (*n*=178), serum klotho levels decreased as a function of higher depressive symptom score (Inventory of Depressive Symptomatology (IDS); *r*=−0.20, *P*=0.007), an association that remained significant after adjusting for age and body mass index (BMI; *B*=−0.17, *P*=0.026; Δ*R*^2^=0.02) (**b**). Women under high chronic stress and experiencing moderate to severe depression (Inventory of Depressive Symptomatology (IDS) ⩾26, *n*=19) had significantly lower levels of klotho compared with women under low stress without depression (IDS 0–12, *n*=57; F(2,99)=4.26, *P*=0.017; pairwise comparison, **P=0.008). In women without depression, *post hoc* comparisons also indicated a trend for lower klotho levels in high-stress women (*n*=26) compared with low-stress women (#*P*=0.079). Analyses were carried out on log10-transformed klotho values. Data are means±s.e.m.

We next determined whether lower levels of klotho were linked with depressive symptoms, a prominent neural correlate of chronic psychological stress.^45^ We found that in all women assessed, lower klotho associated with higher depressive symptoms (*r*=−0.20, *P*=0.007), an association that remained significant after adjusting for age and BMI (*B*=−0.17, *P*=0.026; Δ*R*^2^=0.02; Figure 3a). Because high-stress caregivers experience more depression, we explored whether the combination of being a high-stress caregiver and reporting high depressive symptoms (Inventory of Depressive Symptomatology scores in the moderate and above range ⩾26) had lower levels of klotho compared with high-stress caregivers and low-stress controls who reported minimal levels of depressive symptoms (Inventory of Depressive Symptomatology scores: 0–13). High-stress women with moderate to severe, but not mild, depressive symptoms had significantly lower levels of klotho compared with low-stress women without depression (*P*=0.008; Figure 3b). This statistical difference remained significant after adjusting for age and BMI. In women without depression, there was a trend toward lower levels of klotho in high-stress women compared with low-stress women (*P*=0.079). Thus, depression was linked with lower klotho levels, particularly in high-stress women with moderate to severe depressive symptoms.

## Discussion

Our data reveal that lower levels of klotho are associated with chronic, high-stress and with depressive symptoms in young, otherwise healthy women. In a well-characterized cohort of maternal caregivers, klotho levels were lower in women with high stress and showed an age-related decline. Low klotho was further linked with depressive symptoms, a common maladaptive consequence of stress on the brain. The results of this study show, for the first time, that circulating levels of the longevity hormone klotho are sensitive to environmental influences—and suggest that lower levels may be detrimental to, or serve as a biologic marker for, psychosocial health.

Lower klotho in women with chronically high stress suggests an interactive role for the psychosocial environment in systemic levels of a biological factor that influences longevity,^3, 7, 25^ brain function^12, 26, 48^ and body health.^25^ This is important because it establishes a link between life experience and levels of circulating klotho—in addition to known influences on its levels from genetic *KLOTHO* variation,^12, 23^ aging,^28, 42^ kidney disease^49^ and Alzheimer's disease.^50^

Klotho differences between high- and low-stress caregivers existed when analyzed across all ages, but became clear with greater age, even within a relatively narrow 30-year range from 20 to 50 years. It is interesting to speculate that among adults the effects of chronic stress may have a larger impact on klotho at older ages, although this remains to be tested in future studies. Along these lines, it will be important to determine (1) whether klotho's association with stress drives development and progression of depression, (2) if this relationship depends upon or is synergistic with age, (3) if klotho is a biomarker for either stress or depression and (4) if supplementing low klotho levels could serve as a therapeutic in boosting mental health.

Lower levels of klotho could contribute to stress and its maladaptive correlates such as depression by influencing a myriad of cellular and molecular targets. The function of klotho is complex and multifaceted, with known roles in regulating insulin,^4^ Wnt,^13^ fibroblast growth factor,^14^ nuclear factor kappa B,^22, 51^ tumor necrosis factor-α^22, 52^ and *N*-methyl-D-aspartate receptor signaling.^12^ Importantly, each one of these pathways, which may be dependent or independent of the aging process, can be linked directly or indirectly to stress or depression. Studies are needed to test mechanistically if klotho intersects with these molecular targets to modulate stress or depressive symptoms.

Several methodologic approaches were implemented to ensure validity of our model and findings. Our unique population of young women was assembled to specifically probe effects of high, chronic psychological stress. Caregiving stress, including caring for a child with a developmental disability such as autism spectrum disorder, is a well-established model of chronic stress.^44^ Furthermore, participants were matched on age and BMI and stratified based on a measure of global stress perception, thus optimizing our ability to explore levels of klotho at distinct differences in psychological stress. As we included long-term caregiving, this measure reflects chronic levels of stress. Results may differ if a community sample was used, where sources of stress may be more transient. Of note, when levels of stress perceptions were considered in the present sample, associations between stress and klotho were smaller than group differences. Despite the strength of this design, the generalizability of the data may be limited by its cross-sectional nature, inclusion of primarily Caucasian women and the high level of education and socioeconomic status of this cohort. Thus, longitudinal studies that include men and a broader range of ethnic, socioeconomic and educational backgrounds are warranted. Chronic stress may also associate with klotho levels in children; however, this is yet to be studied. Furthermore, experimental studies, such as behavioral interventions to reduce stress and laboratory-based studies that induce or reduce stress, are needed to infer causation between stress and decreased klotho.

The results of this study raise the possibility that levels of the longevity hormone klotho may be amenable to environmental influences and that lower levels may be detrimental to, or serve as biologic marker for, psychosocial health. Understanding whether lower levels of klotho adversely affect psychosocial well-being, or serve as a biomarker for this measure, may enable us to predict or monitor aging and brain health and identify protective strategies or new therapies for treating stress and depression.

## Figures and Tables

**Table 1:** Demographics and descriptive information of cohort

*Variable*	*Entire sample (*n=*178)*	*Low-stress controls (*n=*88)*	*High-stress caregivers (*n=*90)*
Age (years)	42.0 (5.1)	41.6 (4.6)	42.3 (5.7)
Body mass index	25.5 (5.2)	25.2 (4.7)	25.8 (5.7)
Race (% Caucasian)	75.3	75.0	75.6
Education (% college degree or higher)	84.8	90.7	81.1
			
*Household income per year (%)*			
<$100 000	23.7	14.8	32.6*
$100 000–$149 000	27.1	29.5	24.7
$150 000–$199 000	20.3	25.0	15.7
>$200 000	28.8	30.7	27.0
Perceived stress (PSS scores)	18.7 (5.5)	15.6 (4.4)	21.8 (4.7)*
Depressive symptoms (IDS scores)	15.3 (7.9)	11.7 (5.7)	19.0 (8.1)*
Antidepressant use (% users)	6.2	2.2	10.0*

Abbreviations: IDS, Inventory of Depressive Symptomatology; PSS, Perceived Stress Scale.

Data displayed as means (s.d.'s) or percentages.

*P<0.05 versus controls.

## Notes

Portions of this work are the subject of a provisional patent application held by the Regents of the University of California.
